# Assessing hydrogen as an alternative fuel for rail transport – a case study

**DOI:** 10.1038/s41598-025-90887-3

**Published:** 2025-02-22

**Authors:** S. Rahim Marjani, Shahed Motaman, Hirbod Varasteh, Zhiyin Yang, Jenny Clementson

**Affiliations:** 1 Institute for Innovation in Sustainable Engineering, University of Derby, Derby, UK; 2 School of Engineering, College of Science and Engineering, University of Derby, Derby, UK

**Keywords:** Hydrogen, Rail transport, Environmental impact, NOx emission, Zero carbon transport, Train simulation, Chemical engineering, Civil engineering, Mechanical engineering

## Abstract

Diesel trains play a vital role in the UK’s rail passenger transport. Despite efforts to expand electrification, over 10% of the UK’s rail routes will remain non-electrified. To reduce emissions and phase out diesel trains by 2040, the UK rail network is actively exploring alternative fuels. This paper presents a comprehensive technical, economic, and environmental analysis of converting diesel trains to hydrogen-powered trains using a hydrogen combustion engine for the first time. A simulation-based methodology has been developed to assess train performance, fuel consumption, and emissions for both hydrogen and diesel engines. The developed methodology has been validated by comparing the predictions against the available experimental data and a very good agreement has been obtained. A case study involving British Class 195 diesel-powered regional trains on the Manchester Airport to Barrow-in-Furness route is analysed. The simulation results show that hydrogen-powered trains achieve zero carbon emissions and exhibit similar NOx emissions to diesel, with a similar performance. Over the train’s 30-year lifespan, green hydrogen can reduce CO_2_-equivalent emissions by up to 187.4 kt. The study clearly demonstrates that hydrogen combustion engines offer a practical, mid-term solution for decarbonizing regional rail, with much lower conversion costs compared with fuel cell technology.

## Introduction

The UK’s rail network plays a significant role in the transport system, and compared with other long-distance transport options, it generates lower emissions and is becoming less carbon-intensive as the National Grid decarbonizes^1^. According to the UK’s greenhouse gas emissions report for 2019/20, rail transport contributes 1.4% of total UK transport system emissions^2^. Logan et al.^3^. showed that a change from personal cars towards sustainable electrical or hydrogen-powered public transport was necessary for the UK to meet its net zero emission targets. As rail traffic demand increases, the UK railway network is planning to introduce new low-carbon technologies throughout the passenger and freight rail network, aiming to further reduce emissions and ambitiously phase out diesel trains by 2040. This poses a great challenge in decarbonizing railways, and there is an urgent need to explore alternative fuels for diesel engines. In recent years, there have been two main energy sources in the UK’s railway network, electricity and diesel fuel. Electrification of the railway system has a positive impact on emissions reduction, but it is expensive and requires significant infrastructure investment, which is limited in some areas^4^. On the other hand, diesel fuel is a good source of energy in terms of engine power, good economic performance, and versatility of use on different routes and conditions where electrification is not feasible or limited. However, at the same time, it is also a known source of harmful carbon emissions and particulate matter (PM)^5^.

The UK Department for Transport has set an ambitious goal of removing all diesel-only trains from service and reducing greenhouse gas emissions by 2050^6^. This aspiring target is putting pressure on decision-makers in the railway sector and is inspiring researchers to intensify their efforts to meet this critical challenge. Furthermore, Edward et al.^7^ have presented a comprehensive review of Hydrogen technologies in the UK and suggested that hydrogen can play a crucial role in decarbonizing transport, particularly in areas where electric alternatives face limitations. The UK has committed to low carbon hydrogen^8^ as part of its energy mix and aims to deliver 5GW by 2030 for a mix of use cases, including transport. It is planned to increase the production of low-carbon hydrogen and provide a mix of green electrolytic hydrogen underpinned by an energy strategy^9^, and ‘blue’ hydrogen from reforming with carbon capture and storage, located in industrial clusters. The policy paper acknowledges the challenge of growing supply and demand together across multiple use cases from heating to transport. For rail transport in particular such demand needs to be underpinned by policy and regulation which provides certainty of capital, revenue and incentives for early adopters and safety and quality assurance. The preferred rail use cases will then be those with an economic and technical feasibility case which considers proximity to hydrogen sources and infrastructure on their routes as well as route length and operating parameters^10^.

The railway has a great potential for applying hydrogen as a low-carbon energy. Hydrogen fuel could potentially be used in train engines as an alternative, combustive fuel source to significantly decrease CO_2_ equivalent (CO_2_e) emissions. Hydrogen’s distinctive physical and chemical properties compared with conventional fossil fuels like diesel, make it an environmentally positive alternative for carbon-based emissions, such as CO, CO_2_ and specifically unburned hydrocarbons^11^. However, like diesel, hydrogen engine exhausts still emit NOx. Numerous studies have been conducted in recent years to investigate the performance and emissions of hydrogen direct fuel injection engines, particularly in terms of NOx emissions. A critical review of the application of hydrogen fuel in reducing NOx emissions is presented by Wright and Lewis, which presents an uncertainty about the effect of Hydrogen fuel on overall emissions^12^.

A numerical study by Y.H. Ukpaukure^13^ used the WAVE software to compare alternative fuels, in terms of Brake Specific Fuel Consumption (BSFC), power, NOx, and emissions versus Revolutions per minute (RPM). The hydrogen fuel was found to improve thermal efficiency, and emissions including CO and unburned hydrocarbon (UHC), were reduced by 20% and 50%, respectively, compared with pure diesel. Saravanan et al.^14^ carried out an experimental study on a 4-cylinder naturally aspirated diesel engine with direct-injected hydrogen and diesel fuel and reported that despite higher NOx emissions, a significant reduction of 78% in CO emissions was achieved compared with the diesel fuel alone. Similar findings were reported by other researchers such as Sharma et al.^15^ and Chintala et al.^16^, who observed higher NOx emissions of 25–58% at different engine loads. In these studies, dual-fuel diesel-hydrogen systems were investigated by injecting diesel as a pilot fuel to reach hydrogen’s auto-ignition temperature^17^.

Furthermore, Din et al.^18^ conducted research on the hybrid hydrogen fuel cell propulsion system for the Class 150 passenger train. Their findings demonstrated that using a fuel cell power plant and hydrogen as the primary power source resulted in a substantial reduction of 59% in CO emissions compared with diesel fuel along a specific train route. They developed an in-house code to solve the equations of motion for railway vehicles on a particular route and estimate emissions. Additionally, they demonstrated that hydrogen fuel cell propulsion components could be installed within the existing space of the diesel train Class 150, with no additional space required for hydrogen fuel cell storage tanks. However, fuel cells cannot be considered a solution for Diesel Multiple Units (DMUs) without traction motors, such as the Class 195 diesel train with mechanical transmission, because fuel cells produce electric energy, which traction motors convert into traction force. A similar investigation has been implemented by Sundvor et al.^19^. to evaluate hydrogen fuel cell and battery as the net zero energy sources for Norwegian high-speed passenger trains. They concluded that the suggested solutions could not be used on routes with high-energy demand and a limited timetable.

Most of the research on hydrogen fuel in the railway sector has focused on hydrogen fuel cells, while other studies have centred on the effect of port injection of hydrogen on internal combustion engine behaviour, emissions, and engine performance simulations using computational fluid dynamics (CFD) and experimental measurements. However, a notable gap exists in the utilization of hydrogen direct injection systems in railway combustion engines, which constitutes the focal point of the present study. This study seeks to address this gap by investigating the feasibility and impact of hydrogen direct injection in diesel train engines, specifically focusing on performance and emissions during a real-world journey. The key research question driving this study is: Can hydrogen direct injection offer a viable and cost-effective mid-term alternative to electrification for decarbonizing existing diesel trains? The significance of this issue is underscored by the considerable number of existing passenger trains powered by diesel engines, a number that has grown in recent years. Converting these trains to hydrogen-powered trains with fuel cells is often infeasible due to challenges such as the very high costs of replacing a diesel-mechanic powertrain with a fuel cell-electrical powertrain, maximum power limitation, and technological immaturity. Meanwhile, replacing them with electric trains would also require significant infrastructure investment. To explore the potential of hydrogen direct injection as an alternative fuel, a methodology was developed that integrates a train dynamics model in MATLAB with engine simulation in the WAVE Simulation software. As a case study, the replacement of diesel with hydrogen in a passenger train was analysed using data from an actual journey in the UK. This approach allows for a detailed analysis of the train’s performance and emissions with hydrogen direct injection, offering novel insights into its potential to reduce emissions and improve efficiency without the need for extensive retrofitting or electrification. By focusing on this unique challenge, the study contributes valuable findings to the ongoing efforts of decarbonizing the rail sector, potentially accelerating progress toward the UK’s net-zero goals.

## Methodology

**Fig. 1:**
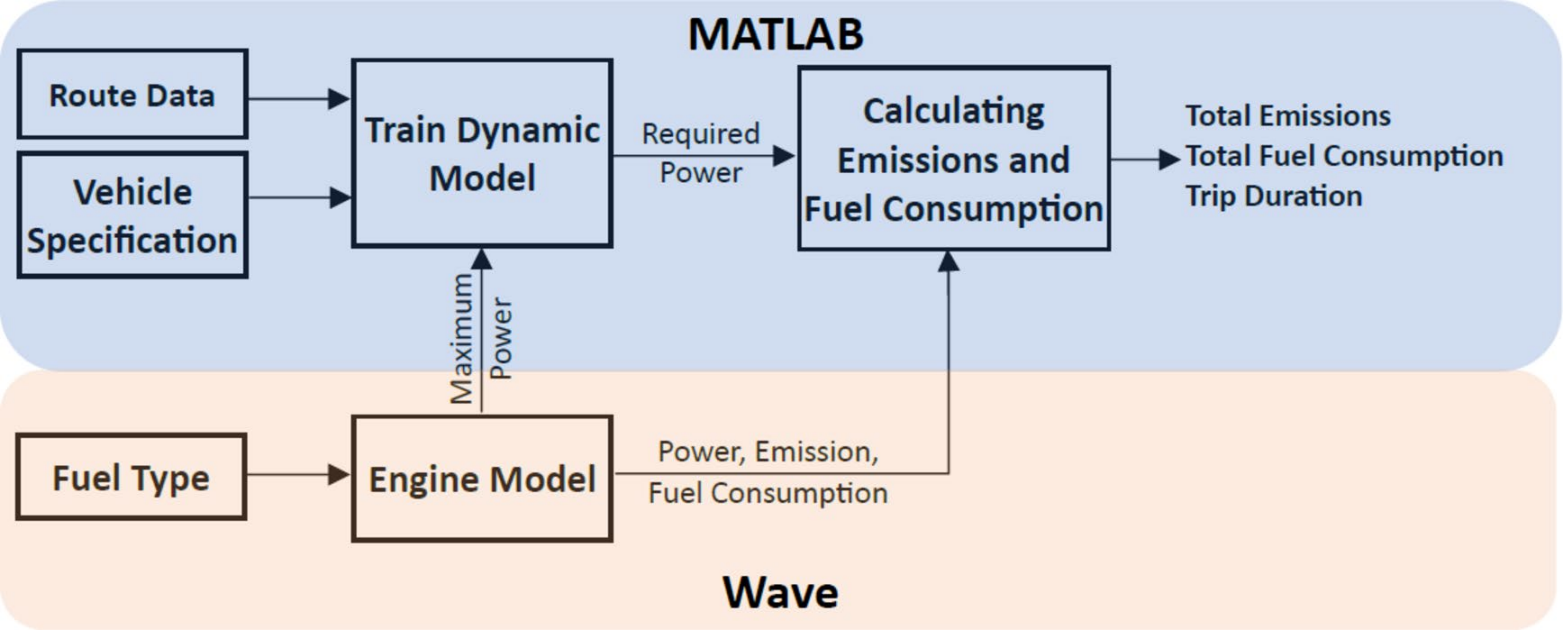
The train dynamics and engine simulation framework.

Figure 1 shows the simulation framework employed in this study. This approach is capable of simulating both train dynamics and engine performance. The train dynamics model has been developed in MATLAB, and the WAVE Realis Simulation (formerly Ricardo Software) software has been employed for the engine simulation.

### Train dynamics model

**Fig. 2:**
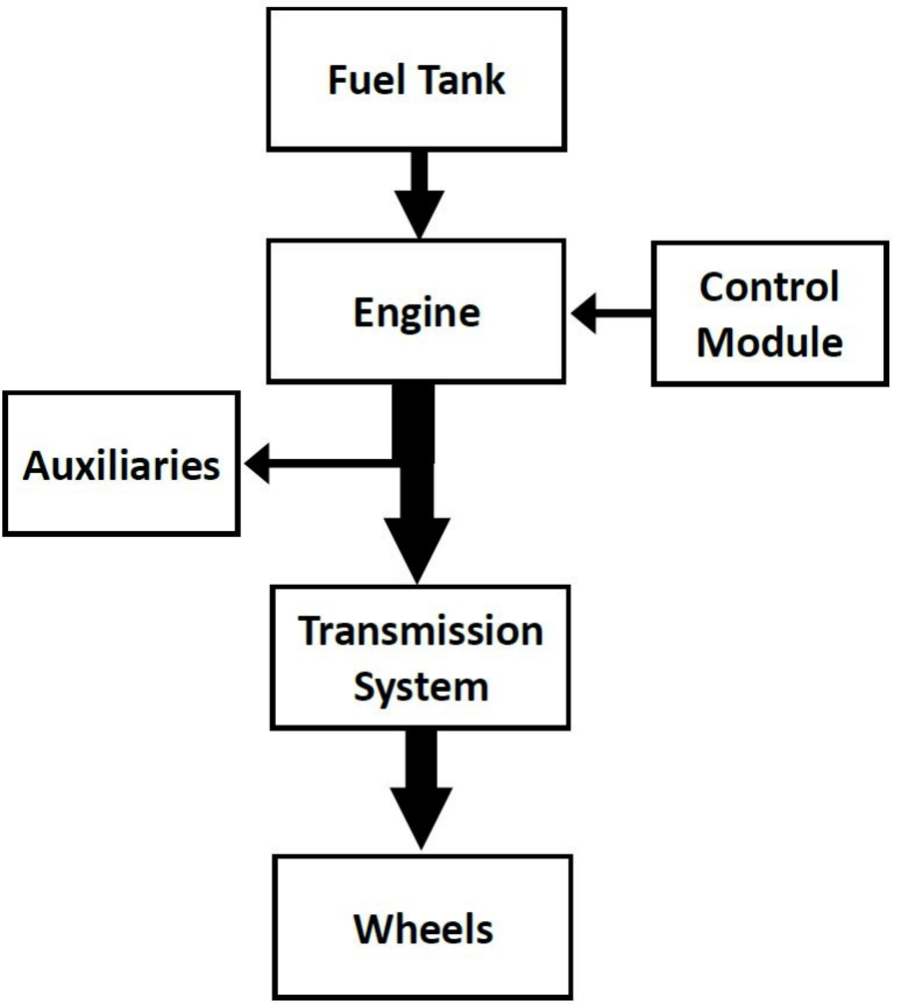
The power modules of the diesel train drive system.

The governing equations for the longitudinal movement of the train are^20^:

1 $$\:{M}_{t}\frac{{d}^{2}s}{d{t}^{2}}=TE-\left({F}_{r}+{F}_{g}\right)$$

2 $$\:{F}_{r}=A+B\left(\frac{ds}{dt}\right)+C{\left(\frac{ds}{dt}\right)}^{2}$$

3 $$\:{F}_{g}={(M}_{v}+{M}_{p})\times g \times \text{sin}\left(\lambda\:\left(s\right)\right)$$

4 $$\:{M}_{t}=\left(1+\alpha\:\right){M}_{v}+{M}_{p}$$

where TE is the traction force, *M*_*t*_ is the total mass, *M*_*v*_ is the train vehicle’s mass, *α* is a rotating components coefficient, *M*_*p*_ is the passengers’ mass, *Fr* is the resistance force calculated using the Davis Eq. (2), *A*, *B*, and *C* are Davis equation coefficients, F_g_ is the force component due to gravity in the direction of train movement when the route is not horizontal and λ(s) represents the gradient of the route (+ for uphill). The traction force can be calculated from^20^:

5 $$\:TE=min\left(\mu\:N,\:\frac{{P}_{wheel}}{V}\right)\:$$

where *µ* is the adhesion coefficient, *N* is the sum of axle loads on the powered axles, *V* is the train’s speed, and *P*_*wheel*_ is the total train power applied to the powered axles defined as^21^:

6 $$\:{P}_{wheel}=\left({{K}_{Notch}\times\:P}_{engine}-{P}_{Aux}\right)\times\:\eta\:$$

where *η* is transmission efficiency K_Notch_ is the power setting (varying between 1 and 7 based on the required power), *K*_*Notch*_ is presented in Table 1, *P*_*engine*_ is the maximum engine brake power, and *P*_*Aux*_ is the required power for auxiliary equipment such as air compressors, lighting, air conditioning etc. Figure 2 shows a schematic diagram of various powers needed, *P*_*wheel*_, *P*_*engine*_ and *P*_*Aux*_ in a train. A control module adjusts the power notch setting in response to the required *P*_*wheel*_ based on the maximum power and route data, such as speed limits. This control module is embedded within the Train Dynamic Model block in Fig. 1. Initially, the notch is set to 7 until the train reaches 99% of its maximum allowable speed or a braking point. Once a train reaches its maximum allowable speed, the power notch is adjusted to sustain a constant velocity. If the train’s speed exceeds the maximum allowable value or it approaches a braking point, the traction force is set to zero, and braking force is applied.

**Table 1:** Standard Notch setting in UK DMU train^21^.

Notch	Idle	1	2	3	4	5	6	7
K_Notch_	0.09	0.18	0.3	0.43	0.57	0.73	0.86	1

### Engine simulation

We have employed the WAVE software to simulate the same engine operating with both diesel and hydrogen fuels. The WAVE software solves the one-dimensional Navier-Stokes equations for mass, momentum, and energy conservation. Additionally, it incorporates sub-models for combustion and emission, enabling the assessment of engine emissions under various fuel types. This study uses a modified diesel engine model to simulate direct injection hydrogen combustion, based on the methodology presented by Antunes et al.^22^. The goal is to evaluate retrofitting feasibility, assuming comparable engine power for both fuels to enable performance and emission comparisons.

### Calculating fuel consumption and emission

The RSSB T1187^21^ report introduces an innovative methodology for accurately estimating emissions from diesel trains, covering a significant portion of active fleets. Initially, notch-based emission factors were established from data obtained through standardized engine tests across a diverse range of diesel trains. To estimate the emissions produced during a train’s journey along a specific route, the route is divided into sections of uniform time intervals. Using information logged by the On-Train Monitoring Recorder (OTMR), engine output power and notch settings are extracted for each section.

The emission for each section is calculated by selecting the appropriate emission factor for the corresponding notch and multiplying it by the engine output power. This process is repeated for all sections along the route, and the results are aggregated to determine the total emissions.

Our study follows a similar approach, with one key distinction: we calculate emission factors and fuel consumption through engine simulations. The engine simulation is conducted under standardized testing protocols in the WAVE environment. Additionally, the required power and notch settings are estimated based on train dynamics simulations for each route section. These values are then substituted into Eq. (7) to calculate emissions and fuel consumption at each time step^21^. When a train stops at a station, the power is reduced to auxiliary power, and the notch is set to “idle”.

7 $$\:Notchbased\:Factor\:\left[\frac{gr}{kWh}\right]\times\:Power\left[kW\right]\times\:time\:step\:\left[h\right]=Emission/Fuel\:\left[gr\right]$$

By integrating the results across all time steps, the total emissions and fuel consumption for the journey can be determined. This simulation-based method enables the study of alternative fuels. In this study, we applied the method to evaluate replacing diesel with hydrogen fuel in passenger trains. However, this approach is flexible and can be adapted to investigate other alternative fuels or modes of rail transport with appropriate modifications to the models.

## Case study

### Vehicle specification

The selected train for this study is a diesel multiple-unit (DMU) passenger train, Class 195, which is used for short and long regional services in the UK. This train represents a wide range of products from CAF, an international train manufacturer, including Class 170, 172, and 196, which have similar powertrains. These DMUs are common across the UK and internationally. This class of trains has been used by Northern Trains Since 2019. The maximum speed of the Class 195 is 100 mph (161 km/h). Given its design, the Class 195 is intended to operate for at least 35 years, lasting even longer than the 2050 target with proper maintenance and overhaul^23,24^. This longevity underscores the significance of minimizing their emissions, as any emission reductions can have a considerable impact on their life cycle environmental impact.

**Table 2:** The British rail class 195 vehicle specification^23,24^.

Specifications	Value	Unit
Tare mass	128.4	tonnes
Coefficient of equivalent rotational mass	0.1	–
Engine power per coach	390	kW
Numbers of powered axles per coach	2	–
Axle arrangement	2’Bo2’	–
*A* ^25^	2.977	kN
*B* ^25^	0.0345	kN/(m/s)
*C* ^25^	0.00474	kN/(m/s)^2^
Auxiliary power	25	kW
Capacity	204	
Passenger mass	80	kg
Maximum speed	100 (161)	Mph (km/h)
Maximum acceleration	0.83	m/s^2^
Transmission efficiency	0.92	–
Adhesion coefficient	0.18	–

Although Class 195 can operate as three- or four-car formations (two-car units coupled), in this study, we focus on a fully occupied train with three-car formations for simulation purposes, with a total power output of 1170 kW (390 kW per coach). Table 2 provides a comprehensive overview of the train’s specifications.

### Route data

The Class 195 operates across various non-electrified routes within the UK’s Northern network. In this study, the Manchester Airport to Barrow-in-Furness route has been selected as a benchmark. The distance between the two stations is 157 km (97 miles), and the train stops at 16 stations. There are approximately 11 direct trains from Manchester Airport to Barrow-in-Furness and 11 direct trains in the opposite direction every weekday. This would reduce to 9 trains on weekends. The first train departs around 5 AM, and the last train departs around 10 PM^26^. The selected route has a typical length, number of stops, and geographic conditions similar to other routes in the UK, making it representative of broader rail transport operations.

**Fig. 3:**
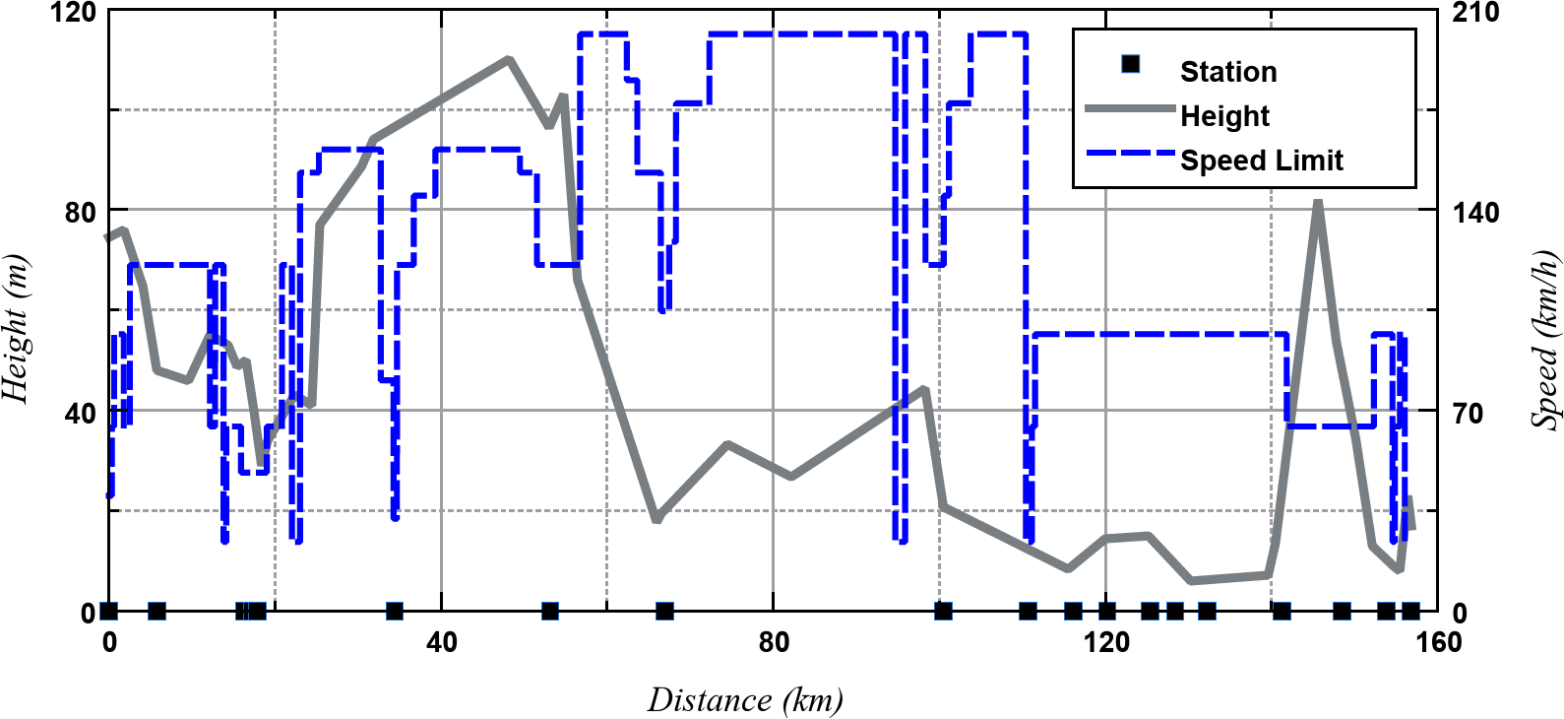
Route profile for Manchester Airport - Barrow-in-Furness^27^, stations, speed limits and height through the route.

The locations of stations, line speed limits, and variations in gradient along the route are depicted in Fig. 3. The speed limit is determined by the minimum of the line speed limit and the maximum train speed (161 km/h). Simulations were conducted in both directions, and the average time spent at each station was assumed to be 30 s.

### Engine model

A heavy-duty I6, 6-cylinder 12.8 L turbo diesel engine was simulated in the WAVE package. This pre-configured WAVE model engine shares remarkably similar specifications to the Class 195, 6 H 1800 passenger train engine. Table 3 provides the full details of the engine model I6 parameters used in this simulation.

**Table 3:** The wave engine specifications used in the simulation^24,28^.

Specifications	Value	Unit
Fuel	Diesel	–
Strokes	4	–
Engine type	6 cylinder-Inline	–
Displacement	12.8	L
Valve/cylinder	4	–
Bore	128	mm
Stroke	166	mm
Maximum RPM range	1800	rpm
Peak torque	2000	Nm
Peak power	390	kW

The engine model comprises distinct elements, including an engine block, cylinders, valves, fuel injector, turbocharger (turbine, compressor, and shaft), ducts, and Y-junctions. The engine’s specifications are designed for direct diesel injection, and the cylinders are connected to the turbocharger for a maximum rotation of 90,000 rpm. The engine simulation input data parameters have been pre-loaded into the model package. The WAVE emission sub-model has been activated to measure NOx and CO emissions as well. Two distinct fuels, diesel and hydrogen, have been chosen for use in this engine. Table 4 presents the fuel properties used in this simulation. Furthermore, due to the higher calorific value of hydrogen compared with diesel fuel, the hydrogen ejection mass flow has been modified accordingly in the WAVE setup.

**Table 4:** Fuel properties used in the WAVE engine model^29^.

Fuel type	Stoichiometric AFR	Lower heating value (LHV) (J/kg)	Density (kg/m^3^) at 1 bar and 300 K	Auto ignition temperature (K)
Diesel	14.22	428e + 05	832	530
Hydrogen	34.07	1.19e + 08	0.082	858

### Validation

#### Diesel fuel

**Fig. 4:**
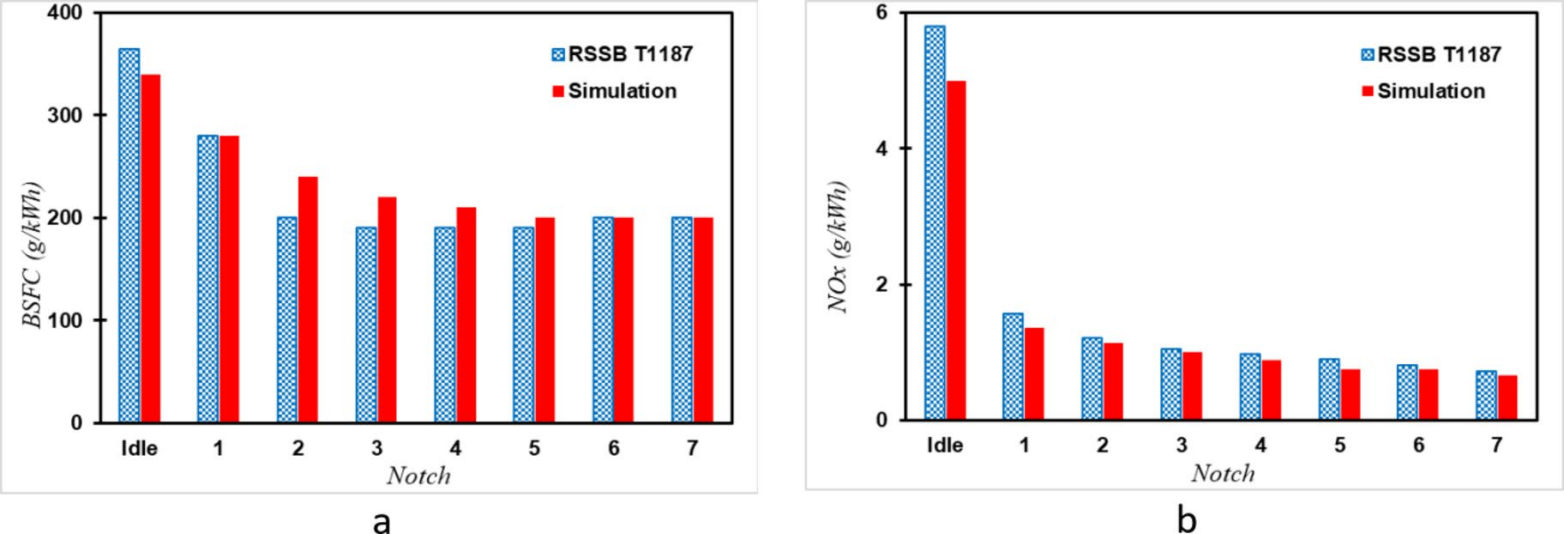
Comparison of simulation results with RSSB T1187 report data for diesel engine in different notches, (**a**) brake specific fuel consumption (BSFC), (**b**) NOx emission factors.

In this section, we validate the diesel engine simulation by comparing it with experimental results. Specifically, we compare the notch-based factors presented in the RSSB T1187 report^21^ to those derived from the engine simulation. In Fig. 4a, the fuel consumption at each Notch derived from the simulation is compared with the corresponding values outlined for a similar engine in the RSSB T1187 report^21^. It’s evident that the simulation findings harmonize with the empirical data across all Notches. Notably, analysis of both the simulation outcomes and empirical observations logged in OMTR for actual trains reveals that the train control system predominantly positions the Notch at 7 or Idle for over 70% of the travel duration. Consequently, the coefficient values associated with these two states bear heightened significance. Additionally, the simulation results closely mirror the experimental data at Notch 7, with a mere 6% deviation observed at the idle state. This underscores the precision and fidelity of the simulation outcomes. The NOx emission factors provided in the RSSB T1187 report are derived from exhaust emissions measured after passing through a Selective Catalytic Reduction (SCR) catalyst, which significantly reduces NOx emissions. However, incorporating an SCR catalyst into the engine simulation isn’t feasible. Instead, we adopt the approach proposed by Wang et al.^30^ to address this challenge. Initially, we simulate the gas temperature at the SCR catalyst inlet and the corresponding NOx emissions. Subsequently, we utilize data from the manufacturer of SCR systems for heavy-duty diesel engines^31^ to determine the SCR conversion efficiency at that temperature. Using this information, we calculate the NOx emissions after the catalyst. Figure 4b illustrates a comparison of NOx emission factors for diesel engines obtained from simulation and the T1187 report. The simulation results indicate a roughly 10% reduction compared with experimental findings. This discrepancy primarily stems from uncertainties and simplifications in estimating the SCR catalyst conversion efficiency.

#### Hydrogen fuel

Substituting diesel with direct hydrogen injection in an engine poses significant challenges in practice. However, numerous laboratory experimental studies have shown its feasibility with proper injector design and control of hydrogen leakage^32^. Moreover, the successful conversion of a diesel engine to a hydrogen engine by JCB^33^, along with its commercial use in heavy-duty vehicles, confirms the potential of hydrogen as an alternative fuel. Nevertheless, existing literature lacks information on the fuel consumption and emissions of such engines. However, having rough estimates for these parameters, albeit imprecise, is crucial for conducting technical and economic studies on converting diesel trains to hydrogen ones. Therefore, this study aims not to design a hydrogen engine but rather to provide estimations of its emission and performance to guide decision-making towards carbon-free rail transportation.

**Fig. 5:**
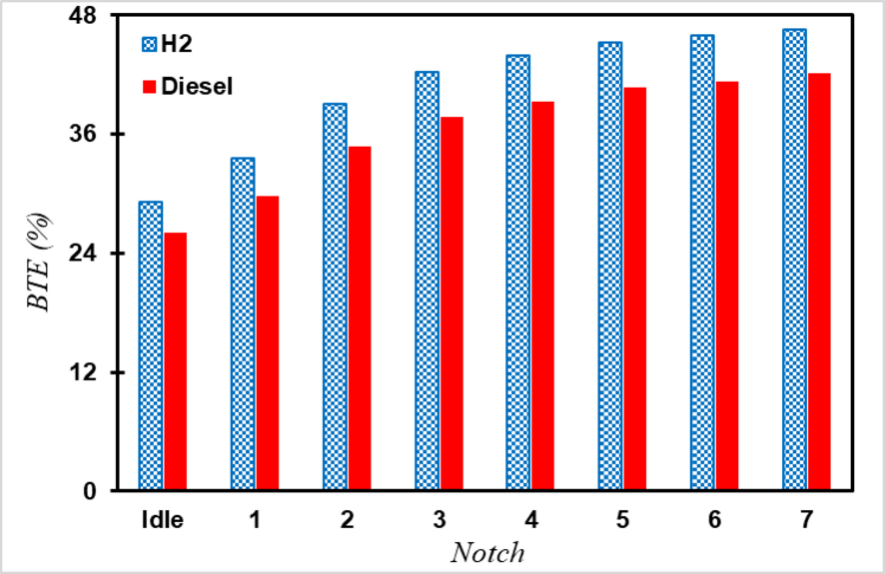
Comparison of brake thermal efficiency of hydrogen and diesel engines.

To convert a diesel engine to a hydrogen engine, we employed the method proposed in laboratory studies by Antunes and et al.^22^. According to this method, increasing the inlet air temperature of the engine up to 120 degrees Celsius is necessary. By implementing the recommended modifications and fuel type, simulations were conducted under similar conditions. Additionally, the fuel amount was adjusted to match the engine output power to that of a diesel engine. As shown in Fig. 5, the hydrogen-fuelled engine exhibits better thermal efficiency compared with the diesel engine, with improvements of 3.1% at idle and 4.5% at Notch 7. These improvements can be attributed to the rapid and complete mixing of hydrogen gas with uniform distribution inside the cylinder, rapid hydrogen combustion, and increased oxygen levels due to the higher air-to-fuel ratio in hydrogen engines, facilitating a more complete combustion process.

**Fig. 6:**
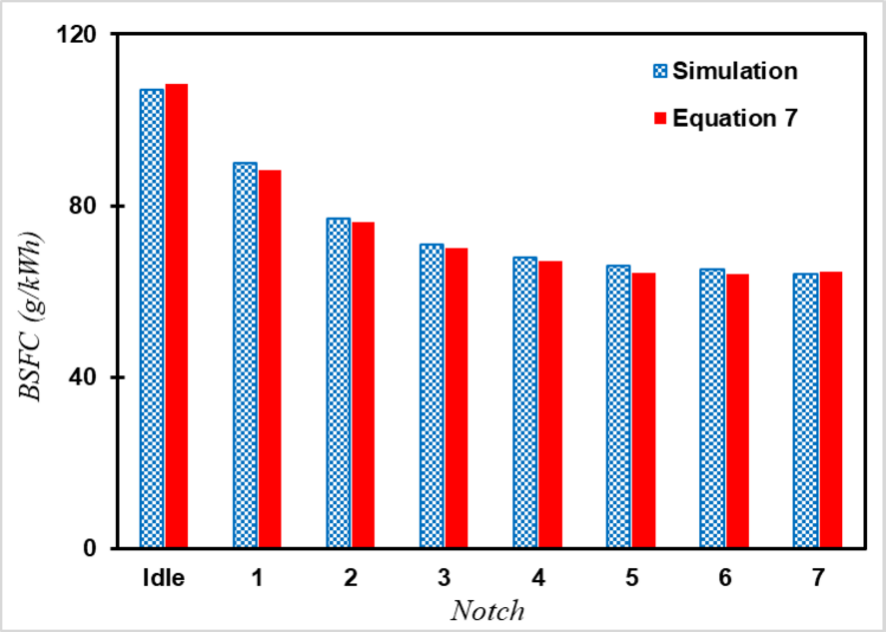
The BSFC of the engine with Hydrogen fuel in different Notches.

Given that the engine output power remains constant with both fuels, hydrogen fuel consumption can be estimated based on the definition of thermal efficiency (BTE) and BSFC using Eq. 8^34^. Figure 6 illustrates the fuel consumption at each Notch based on Eq. 8, and simulation, showing perfect alignment.

8 $$\:{\left(BSFC\right)}_{{H}_{2}}=\frac{{\left(LHV\right)}_{Diesel}}{{\left(LHV\right)}_{H2}}\times\:\frac{{\left(BTE\right)}_{Diesel}}{{\left(BTE\right)}_{H2}}\times\:{\left(BSFC\right)}_{Diesel}$$

**Fig. 7:**
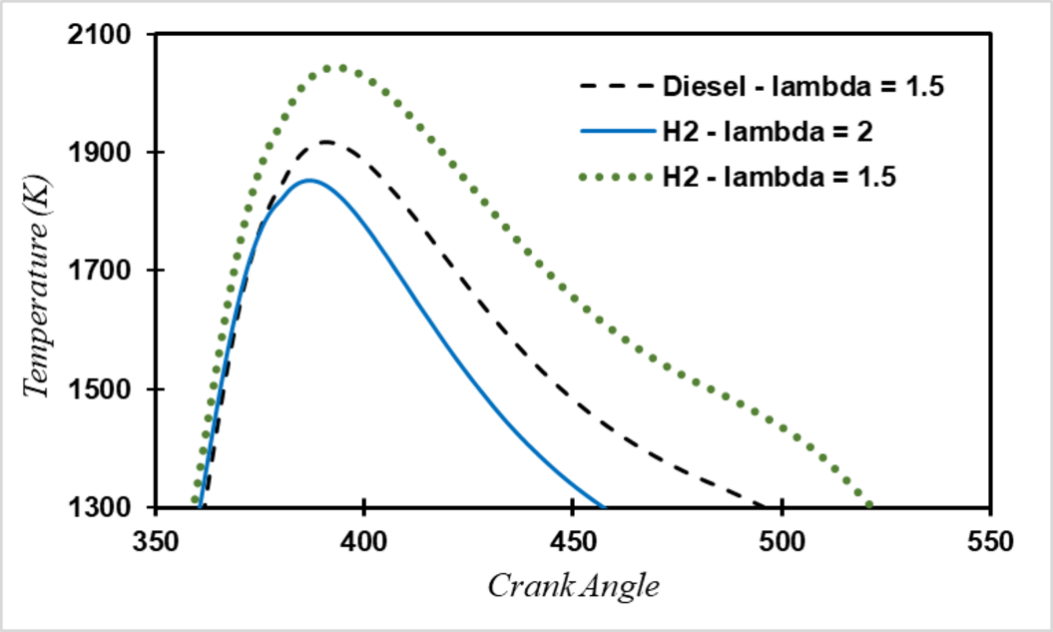
In-cylinder Temperature at full load and rpm 1600.

Initially, the fuel-to-air ratio in the simulation was assumed to be similar to diesel fuel for calculating NOx emission factors. However, this resulted in a significant increase in maximum cylinder temperature and consequently a 20 to 30% rise in NOx emissions, as seen in Fig. 7. When hydrogen burns with a very hot flame, the normally stable molecules split in the reaction, and nitrogen in the air reacts with oxygen^35^:

9 $$\:N+{O}_{2}\to\:NO+O$$

10 $$\:{N}_{2}+O\to\:NO+N$$

**Fig. 8:**
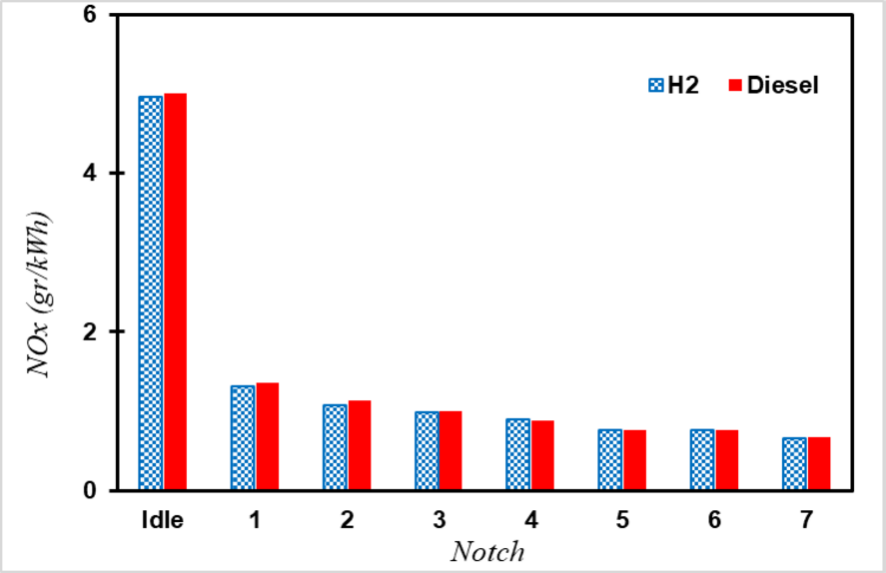
Comparison of NOx emission coefficients of hydrogen and diesel engines.

These reactions occur at temperatures above 1,400 degrees Celsius, the threshold temperature. Since such high temperatures are impractical and can damage the cylinder, the fuel-to-air ratio was adjusted to maintain a maximum cylinder temperature like that of diesel mode. Despite the faster cooling of the engine during hydrogen combustion, NOx emission factors remained similar to those of diesel due to increased oxygen levels in the cylinder, leading to a higher likelihood of NOx formation. Furthermore, in hydrogen engines, there is no need for a catalyst to remove CO and PM, enabling the catalyst to be installed closer to the engine, with exhaust gases entering directly into the catalyst. By implementing these changes in the simulation, the inlet temperature to the SCR catalyst for hydrogen fuel was similar to diesel fuel, resulting in NOx emission coefficients for hydrogen fuel like those for diesel as shown in Fig. 8.

Moreover, it’s worth noting that numerous studies have shown that hydrogen injection into the exhaust gas enhances the performance of the SCR catalyst. The presence of hydrogen in the engine exhaust gas is unavoidable due to leakage and incomplete combustion, which can enhance the catalyst’s effectiveness. This underscores the importance of studying catalysts specifically designed for hydrogen engines alongside hydrogen engine development.

## Results and discussion

The validated model has been used to simulate the journey of Class 195 trains on the Manchester Airport to Barrow-in-Furness route. The calculated total journey time is 131 min for the train powered by both fuels, including a 30-second dwell time at each station. We assumed that the train runs as fast as possible, so the calculated time represents the minimum possible duration for the trip. The reported time on tickets is 144 min which is 9% higher than the calculated time. This difference is expected and acceptable since the train drivers do not apply maximum traction and brake power and there may be longer dwell times in busy stations.

### Train performance

**Fig. 9:**
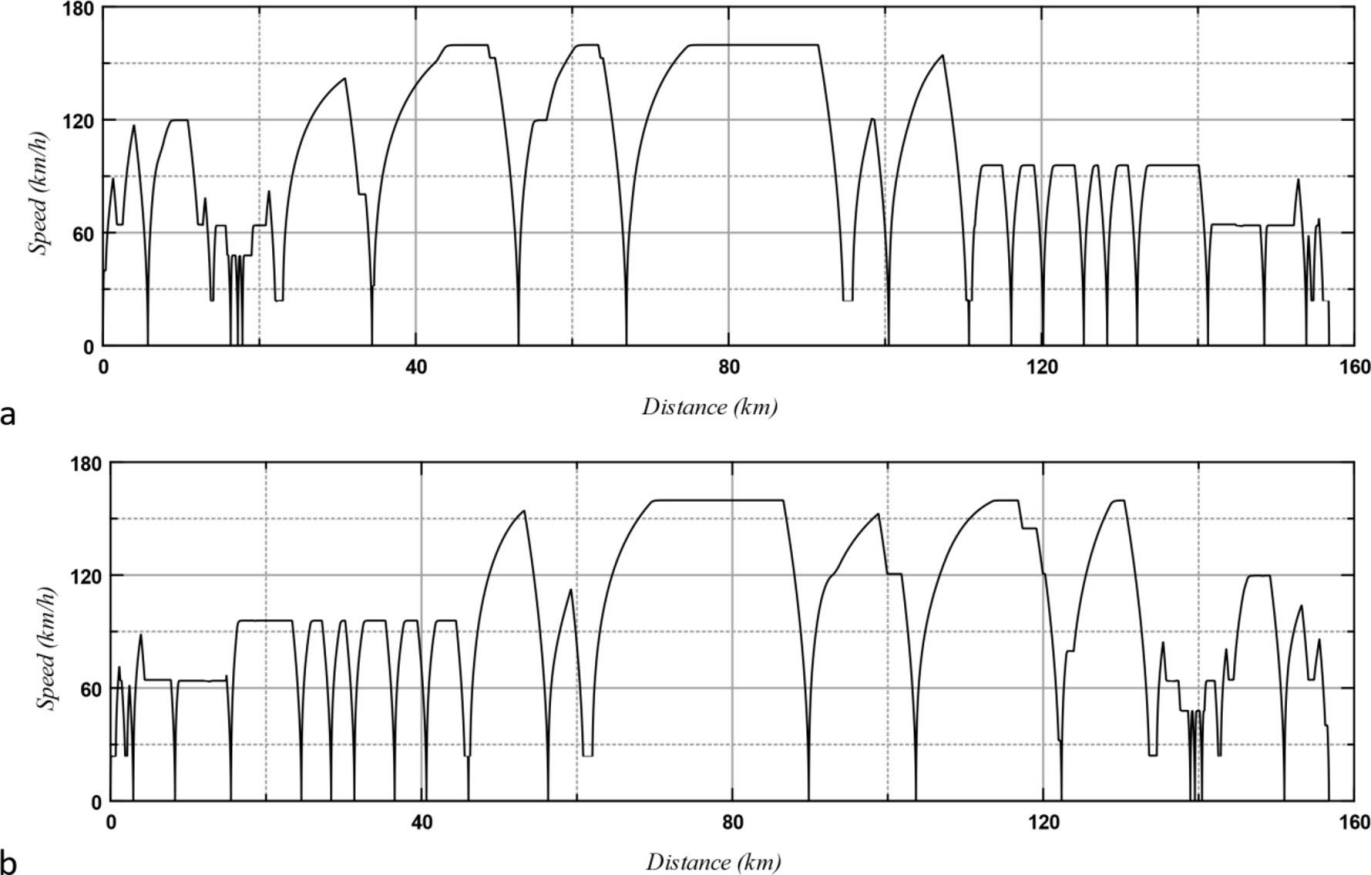
The train speed throughout the route: (**a**) outward and (**b**) return journeys.

The simulated speed of the train is shown in Fig. 9. The velocity of the train is significantly lower than its maximum speed throughout most of the journey. This is due to the frequent stops at stations and the low-speed limitations on many sections of the route. The train can only reach its maximum speed on a limited number of segments along the route.

### Engine power and Notch setting

**Fig. 10:**
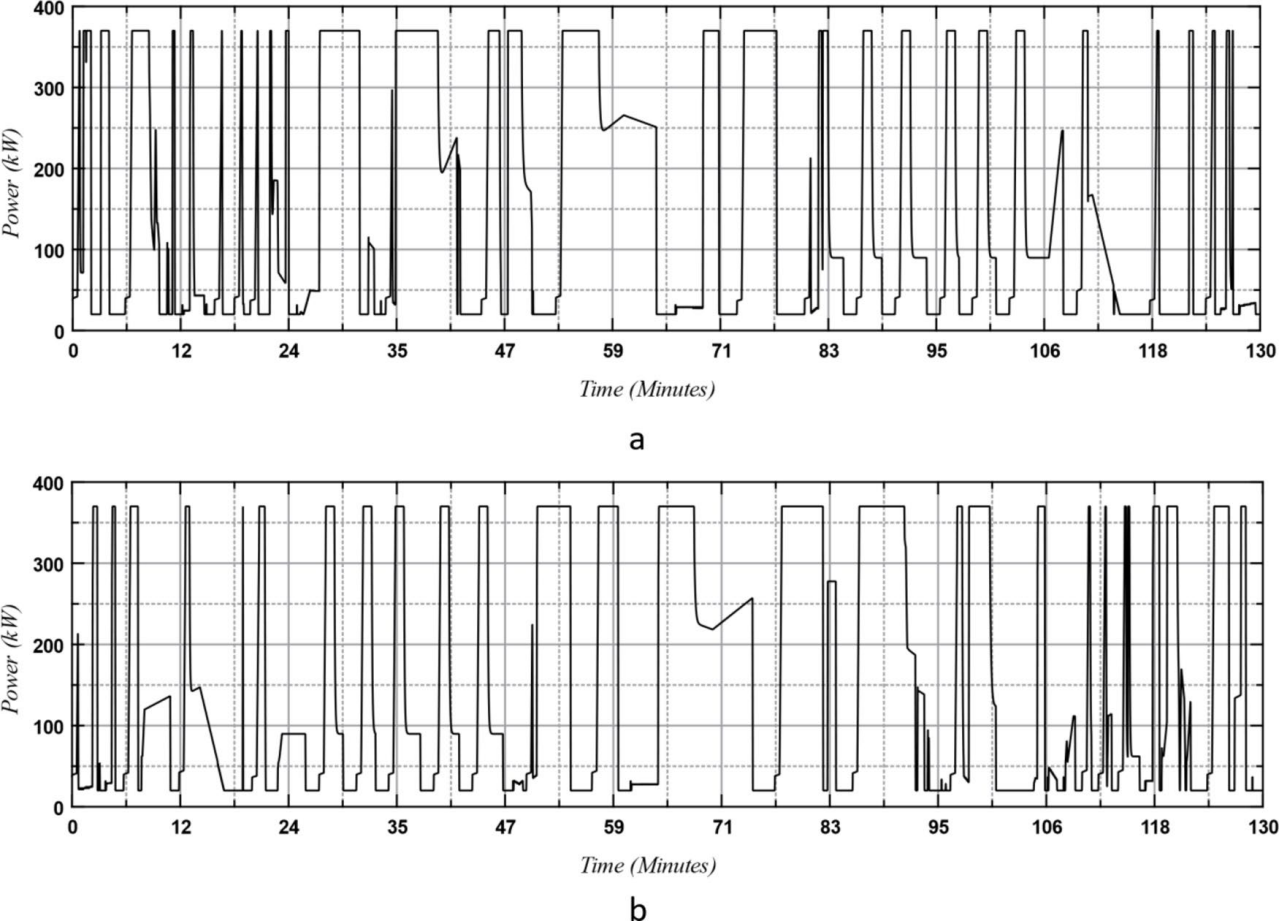
The engine power: (**a**) outward and (**b**) return journeys.

The output power from the engine train is shown in Fig. 10 for both the outward and return journeys. Some of this power is allocated to operate the auxiliary equipment, which remains active even when the train is stopped. Therefore, the power drawn from the engine is not zero at stations.

**Fig. 11:**
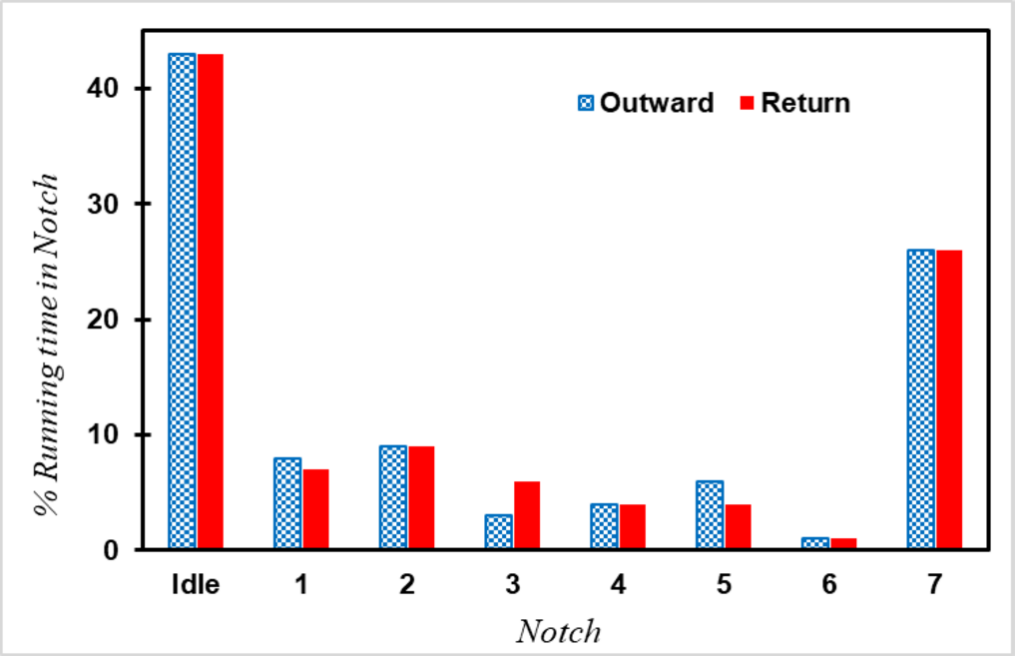
Percentage of running time in each Notch.

Figure 11 depicts the percentage of running time in each Notch through both Outward and Return Journey. As can be seen, because of numerous stops throughout the journey, 44% of the time the train is in idle mode, during brake, coasting and stopping in stations, and 26% in Notch 7. This type of operation with lots of braking and acceleration causes increasing emissions and fuel consumption.

### Total fuel consumption and emissions

Although hydrogen produces NOx and it produces zero carbon emissions, as there is no particulate matter, including carbon dioxide, carbon monoxide, and hydrocarbons (HC). The heating value of diesel is significantly lower than that of hydrogen. The results related to total emissions and fuel consumption are presented in Table 5. The NOx emissions were calculated using engine simulation results, while the CO_2_ emission was estimated based on total fuel consumption and the method presented in^36^. Since hydrogen does not contain any hydrocarbons, CO_2_ emissions are zero.

**Table 5:** Comparison of emission and fuel consumption for diesel and hydrogen-powered trains.

Fuel type	Outward journey			Return journey		
	CO_2_ (kg)	NOx (kg)	Fuel consumption (kg)	CO_2_ (kg)	NOx (kg)	Fuel consumption (kg)
Diesel	636	0.92	201 (242 L)	649	0.91	205 (248 L)
Hydrogen	0	0.93	65	0	0.91	66

It is evident from Table 5 that the hydrogen-powered train produces zero carbon emissions compared with the diesel-powered but there is similar NOx emission for the reasons outlined in Sect. 3.4.2. So, the NOx reduction must be a core focus for inclusion in research related to developing hydrogen engines. NOx production is closely related to the combustion temperature and the emissions increase upon exceeding a threshold temperature. Hence, the control of temperature in burning is critical to minimise the NOx emission for hydrogen fuel and one feasible way is to operate hydrogen combustion engines at lean conditions (a higher air-fuel ratio than the stoichiometric ratio). Moreover, a high level of exhaust gas recirculation can be used as an alternative method for reducing NOx emissions. In addition, designing a new SCR catalyzer specifically for hydrogen engines is vital to minimize NOx emissions.

## Technical, economic and environmental analysis of hydrogen multiple units (HMU) train

### Engine conversion

To safely and efficiently convert a diesel engine to burn hydrogen, several engine components must be replaced or modified. Key components involved in this conversion include hydrogen-compatible fuel injectors, turbocharging and air management systems, and modifications to the engine control unit (ECU). Additionally, changes to the cylinder head, valves, and intake/exhaust systems are required to accommodate hydrogen’s unique combustion characteristics, which involve faster burn rates and different temperature profiles compared with diesel. Upgrades to the cooling system are also essential to manage the higher combustion temperatures associated with hydrogen fuel. Furthermore, hydrogen-specific safety measures, such as venting and leakage detection systems, must be integrated to ensure operational safety. Testing and calibration are critical to ensuring that the converted engine meets performance and emissions standards^37–39^.

The costs associated with converting a diesel engine to a direct hydrogen fuel injection system are estimated to be up to £120,000 per car. These figures are consistent with estimates from reports on heavy-duty engine retrofits for alternative fuels, including hydrogen^40^. This cost could be reduced in the near future by increasing the number of approved technologies for hydrogen engines.

### Hydrogen storage and refuelling station

One of the main challenges in utilizing hydrogen as a fuel for trains is determining an efficient method for storing and refuelling hydrogen onboard. Xu et al.^41^ provided a comprehensive analysis of various hydrogen storage technologies for railway applications. Currently, high-pressure tanks are the most widely used and approved technology for hydrogen storage in trains. For instance, Alstom’s first commercial hydrogen fuel cell passenger train utilizes high-pressure tanks with a hydrogen capacity of 130 kg per wagon^42,43^. Similarly, in China, the CRC tested a shunting locomotive with a hydrogen capacity of 260 kg per locomotive, both using 350 bar pressure tanks^44^. The automotive industry also employs high-pressure tanks for hydrogen storage, but typically at a higher pressure of 700 bar. This higher pressure offers a 50% improvement in volumetric capacity compared with 350 bar systems, allowing for 1.5 times more hydrogen to be stored in the same volume. However, it is about 25% more expensive^45^.

For intercity trains like Class 195, there is less of a volume constraint compared with passenger cars. Therefore, in this study, a 350 bar storage system is considered, as it offers a safer and more cost-effective solution while meeting the train’s storage needs.

It is assumed that each train will have a total hydrogen storage capacity of 270 kg (90 kg per car), sufficient for a journey of 650 km, which is more than enough for two return trips between Manchester Airport and Barrow-in-Furness. The method presented by Xu et al.^41^ was used to estimate the required space, weight, and cost of this storage system. Each storage system will require 5,100 L of space per car. Removing the diesel tank provides up to 1,600 L of space and any additional space can be accommodated by installing hydrogen tanks on the roof, similar to the design of the Alstom hydrogen train^43^.

The weight of the hydrogen storage system will be approximately 1,700 kg per car, which is almost equivalent to the weight of a full diesel tank. Therefore, replacing the diesel tank with hydrogen storage will not significantly change the total weight of the train. The estimated cost for the storage system is £36,000 per car.

Each train will have a 40-minute refuelling window, including 25 min for refilling and 15 min for manoeuvring. A refuelling station with three dispensers, each with a refilling rate of 3.6 kg/min, will be required, which is achievable with current technology. To cover the timetable presented in Sect. 3.2, four 3-car hydrogen trains will be needed. Each train will complete three full return journeys and consume a total of 393 kg of hydrogen per day. As a result, the refuelling station must have a daily capacity of 1,600 kg to support all four trains. A comprehensive study on hydrogen refuelling stations is presented by Tobias Eißler et al.^46^. According to this study, a station of this capacity, classified as an XL station, would require an initial capital investment of £2.8 million to build.

### Life cycle emission

The life cycle of a fuel includes various stages, such as fuel extraction or production, transportation, and end-use combustion. A standard method for assessing life cycle emissions is through the calculation of CO_2_ equivalents (CO_2_e), where 1 kg of CO_2_e represents the environmental impact equivalent to 1 kg of CO_2_ emissions.

Hydrogen can be produced using different methods and energy sources, which results in its classification into various “colours.” These colours are essential when assessing the life cycle emissions of hydrogen. The primary classifications are grey, blue, and green hydrogen. Grey hydrogen is produced from fossil fuels without carbon capture, utilization, and storage (CCUS) and has the highest production emissions. If CCUS is incorporated into the hydrogen production process, the hydrogen is classified as blue hydrogen, which has reduced emissions. Green hydrogen, the cleanest form, is produced through electrolysis powered by renewable energy sources such as wind^47^. Table 6 presents the CO_2_e coefficients for various fuels.

**Table 6:** CO_2_e coefficients for various fuels.

Life cycle stage	Diesel^36^ (kg CO₂e per kg diesel)	Green hydrogen^48^ (kg CO₂e per kg H_2_)	Blue hydrogen^48^(kg CO₂e per kg H_2_)	Grey hydrogen^48^ (kg CO₂e per kg H_2_)
Production/transport	1.1	1	4.4	9.7
End-use combustion (CO_2e_)	3.23	0	0	0
Overall LCA emission	4.33	1	4.4	9.7

As shown in Table 7, for hydrogen to be considered a truly green energy source for combustion engines, it is crucial to increase the production of green hydrogen and focus on minimizing NO_x_ emissions generated during the combustion process. The CO_2_e for the entire life cycle of the train has been calculated based on the following assumptions: there are four trains, each completing three return journeys per day, operating for 320 days per year. The remaining operational life of the trains is 30 years. Using this data, along with the information presented in Tables 5 and 6, the total CO_2_e for each type of fuel has been calculated and is presented in Table 7.

**Table 7:** Overall LCA emissions of various fuels.

Fuel	Total CO_2_e (million kg)	Reduction compared with diesel
Diesel	202.5	
Grey hydrogen	146.4	27.7%
Blue hydrogen	66.4	67.2%
Green hydrogen	15.1	92.5%

It is evident that replacing diesel with hydrogen in train combustion engines results in a significant reduction in life cycle emissions. The reduction is estimated to be 187.4 kt CO_2_e for green hydrogen, 136.1 kt CO_2_e for blue hydrogen, and 56.1 kt CO_2_e for grey hydrogen. Future advancements in reducing NOx emissions from hydrogen combustion engines, along with improvements in the CO_2_e of hydrogen production processes, could further enhance these reductions.

The fuel cell has at least a 15% better efficiency than a hydrogen combustion engine and it doesn’t produce any NO_x_, hence it is a cleaner solution for using hydrogen in rail transport. However, the overall cost of converting a DMU Class 195, with a maximum speed of 160 km/h, to a hydrogen multiple unit (HMU) — including engine upgrades, storage system, design, installation, and approval processes — is estimated to be around £200,000. This cost is significantly lower than the £3.2 million estimated by Din and Hillmansen^18^ for replacing the powertrain of a DMU Class 150 (maximum speed 120 km/h) with a fuel cell electric powertrain, which also has a lower power output. It’s important to note that the average price of a new DMU Class 195 is approximately £1.75 million. Furthermore, fuel cell technology is newer than combustion engine technology and would require substantial investment in upgrading maintenance depots and retraining personnel. In addition, in Diesel Multiple Units (DMUs) such as Class 195, all powertrain components are mechanical, with no electrical traction motors. As a result, converting the power source from a combustion engine to a fuel cell would require significant modifications. Most components of the control unit and bogies would need to be replaced to transition from a purely mechanical powertrain to an electrical one, including the addition of traction motors. This process is comparable to designing a completely new train. Therefore, while fuel cells are not yet a viable option for achieving lower emissions in rail transport, hydrogen combustion offers an affordable and practical mid-term solution.

Hydrogen combustion engines have increasingly become a popular research area. Mohammadi et al.^49^ reported that the injection of hydrogen directly into the cylinder during the compression stroke could enhance the efficiency of a hydrogen internal combustion engine (H2ICE). Several studies^50–53^ have shown that the performance and NOx emissions of hydrogen combustion engines are affected by many factors such as compression ratio, injection pressure, and injection timing, to name a few.

## Conclusions

This study provides a comprehensive technical, economic, and environmental analysis of applying hydrogen combustion engines to intercity passenger rail transport for the first time. A simulation method has been developed to estimate the performance and emissions of both hydrogen- and diesel-powered trains. The developed method integrates engine simulation data from the WAVE software for both diesel and hydrogen engines with train dynamic simulations in MATLAB. The simulation results have been validated against experimental data from previous studies, ensuring the robustness of the model.

The findings indicate that hydrogen engines exhibit higher brake thermal efficiency than diesel engines across all engine loads. Hydrogen also offers the advantage of zero carbon emissions, while producing NOx emissions comparable to diesel. However, this emphasizes the need for further research into catalytic converters specifically designed for hydrogen engines to minimize NOx emissions.

The study simulated the operation of Class 195 DMU trains on a real regional route between Manchester Airport and Barrow-in-Furness, calculating total fuel consumption and emissions for both diesel and hydrogen-powered journeys. Technical, economic, and environmental analyses of converting four 3-car DMU trains on this route to hydrogen-powered (HMU) trains were also conducted. The estimated cost of conversion is approximately £200,000 per car, which is significantly lower than the cost of converting to fuel cell technology, which would require additional investments in maintenance facilities and personnel training.

Each train requires a hydrogen storage capacity of 270 kg, sufficient for a 650 km journey, with hydrogen stored in 350-bar tanks. This storage system, modelled after existing commercial solutions, is both affordable and feasible within the available space on the train. Refuelling infrastructure, capable of supporting multiple trains daily, is technologically viable but would require further investment. The study also evaluated the life cycle emissions of grey, blue, and green hydrogen. Green hydrogen provides the largest emissions reduction over the train’s operational lifetime, cutting emissions by 187.4 kt CO_2_e. As advancements in hydrogen production and NOx control for hydrogen engines emerge, these benefits are expected to grow.

Given hydrogen’s significant potential as a clean fuel, increased support and further research are urgently needed. Stronger policy measures should be implemented to encourage the adoption and development of hydrogen technologies. In terms of future research, several promising areas have been identified:

- Hydrogen Storage: Research should focus on increasing the hydrogen storage capacity for trains and other applications.
- 3D CFD Modelling: The development and optimization of a 3D Computational Fluid Dynamics (CFD) model for simulating direct hydrogen fuel injection in heavy-duty diesel engines, and the availability of this model for applied systems research, will be crucial. This model can help optimize fuel injection strategies, turbocharging systems, and combustion parameters to improve efficiency while minimizing emissions.
- Emission Control: Advanced emission control technologies such as lean-burn combustion and exhaust gas recirculation (EGR) should be explored to reduce pollutants. Additionally, the development of catalytic converters specifically designed for hydrogen engines is essential for minimizing NOx emissions.
- Hybrid Systems: Research into hybrid train systems using battery power at low speeds and hydrogen power during high-demand conditions could further enhance efficiency. Regenerative braking combined with hydrogen-powered generators could also be explored as a way to recharge batteries and improve overall sustainability.
- Many parameters cannot be fully investigated using simulation methods, such as CO, CO₂, and HC emissions in the exhaust, which are associated with the combustion of lubricating oil in the piston-ring-piston sleeve system. Therefore, it is essential to conduct experimental studies to explore these unknown aspects and measure emissions accurately.

## Data Availability

The datasets used and/or analysed during the current study are available from the corresponding author upon reasonable request.
